# Selective protein kinase C inhibition switches time-dependent glucose cardiotoxicity to cardioprotection

**DOI:** 10.3389/fcvm.2022.997013

**Published:** 2022-09-07

**Authors:** Sean Brennan, Simona Esposito, Muhammad I. M. Abdelaziz, Christopher A. Martin, Samir Makwana, Mark W. Sims, Iain B. Squire, Parveen Sharma, Amy E. Chadwick, Richard D. Rainbow

**Affiliations:** ^1^Department of Cardiovascular, Metabolic Medicine and Liverpool Centre for Cardiovascular Sciences, Institute of Life Course and Medical Sciences, University of Liverpool, Liverpool, United Kingdom; ^2^Department of Cardiovascular Sciences, University of Leicester, Glenfield General Hospital, Leicester, United Kingdom; ^3^Leicester NIHR Biomedical Research Centre, Glenfield General Hospital, Leicester, United Kingdom; ^4^Department of Pharmacology and Therapeutics, Institute of Systems, Molecular and Integrative Biology, Liverpool, United Kingdom

**Keywords:** glucose, cardiotoxicity, cardioprotection, hyperglycaemia, protein kinase C (PKC), time-dependent

## Abstract

Hyperglycaemia at the time of myocardial infarction has an adverse effect on prognosis irrespective of a prior diagnosis of diabetes, suggesting glucose is the damaging factor. In *ex vivo* models of ischaemia, we demonstrated that deleterious effects of acutely elevated glucose are PKCα/β-dependent, and providing PKCα/β are inhibited, elevated glucose confers cardioprotection. Short pre-treatments with high glucose were used to investigate time-dependent glucose cardiotoxicity, with PKCα/β inhibition investigated as a potential mechanism to reverse the toxicity. Freshly isolated non-diabetic rat cardiomyocytes were exposed to elevated glucose to investigate the time-dependence toxic effects. High glucose challenge for >7.5 min was cardiotoxic, proarrhythmic and lead to contractile failure, whilst cardiomyocytes exposed to metabolic inhibition following 5-min high glucose, displayed a time-dependent protection lasting ∼15 min. This protection was further enhanced with PKCα/β inhibition. Cardioprotection was measured as a delay in contractile failure and K_ATP_ channel activation, improved contractile and Ca^2+^ transient recovery and increased cell survival. Finally, the effects of pre-ischaemic treatment with high glucose in a whole-heart coronary ligation protocol, where protection was evident with PKCα/β inhibition. Selective PKCα/β inhibition enhances protection suggesting glycaemic control with PKC inhibition as a potential cardioprotective therapeutics in myocardial infarction and elective cardiac surgery.

## Introduction

Elevated blood glucose at the time of acute myocardial infarction (AMI) has been shown to worsen prognosis irrespective of a prior diagnosis of diabetes (1–4). Elevated admission blood glucose, fasting glucose and average glucose levels during hospitalization have all been associated with increased mortality in acute coronary syndrome (ACS) patients (4–6). Combined, these data indicate that elevated glucose over a short time frame can worsen ACS prognosis and may explain why rapid glucose lowering treatment can be beneficial. Consequently, The American Heart Association (7), European Cardiovascular Society (8), and The National Institute for Health and Care Excellence (NICE, United Kingdom) (9) all recommend careful monitoring and limiting of both hyper- and hypoglycaemia in AMI patients.

Our, and others, previous findings show a damaging effect of enhanced glycolytic metabolism, via *acutely* elevated extracellular glucose, on cellular function in otherwise physiological conditions and abolition of cardioprotection against simulated ischemia, through activation of protein kinase C (PKC) α and β isoforms (10–17). The clinically negative impact of acute hyperglycaemia during ACS is, therefore, supported by non-diabetic cellular, whole tissue *ex vivo, in vivo*, and clinical data (2, 9). The 2021 NICE guidelines for acute coronary syndromes recommend further research in optimal management of hyperglycaemia in ACS. In this manuscript we investigate the time-dependence of the damaging effect of enhanced glycolytic metabolism and whether inhibition of protein kinase C (PKC) α and β isoforms can reduce the onset of these damaging effects.

Whilst glucose-insulin-potassium infusions have been a mainstay of hospital treatments for myocardial infarction, the benefits of this have not been always clear (18–21). The most effective use of this treatment appears to be as a cardioprotective *pre-treatment* in elective surgery (22–24). We have previously shown that glucose has acute cardiotoxicity, in this context referring to an increased proarrhythmicity with enhanced cell death and infarct size in cell and animal models (14, 17, 25). This is not, however, the complete story, where the duration of exposure to an elevated glucose has significant ramifications for cellular outcome. Our data suggests there is a “temporal cliff-edge” between elevated glucose being protective and toxic to cardiomyocytes.

Here, we show that exposure to elevated glucose for >7.5 min decreased contractile recovery following a period of metabolic inhibition, suggesting that this triggers a period of enhanced glycolytic metabolism that is potentially cardiotoxic. If the duration of high glucose challenge was less than 5 min, it did not appear to cause cardiotoxicity and *enhanced* cell survival in a metabolic inhibition protocol, indicating cardioprotection. These data led to the hypothesis that brief (<5 min) challenges of elevated glucose would impart cardioprotection, analogous to the brief ischaemic intervals used in ischaemic preconditioning and as previously reported with ethanol pre-treatment (26, 27). Conversely, longer duration of glucose perfusion imparted toxicity, suggesting a tipping point where the damaging PKCαβ-dependent toxicity dominates. Finally, selective PKCαβ inhibition abolished the longer duration toxicity of elevated glucose and further improved cardioprotection.

## Materials and methods

### Ethical approval

All methods were carried out in accordance with relevant guidelines and regulations. Adult male Wistar rats (300–400 g, 12–14 weeks old, *n* = 94, bred in-house at the University of Leicester), were killed by concussion and cervical dislocation. Animals were maintained in 1,800 cm^2^ Tecniplast cages, using BED01/8 dates and corn cob for bedding, housed in individually ventilated caging (specific-pathogen-free). The number of cage companions for the animals of 300–400 g is 5 based on a calculation of 350 cm^2^ being required for each rat of that size. The care and death of the animals confirmed to the requirements of the UK Animals (Scientific Procedures) Act 1986 (SI 2012/3039). All procedures were approved by the University of Leicester animal welfare ethical review board (AWERB_2018_44). The ARRIVE guidelines for reporting experiments involving animals have been followed in this study (28).

### Solutions

All perfusing solutions were based on a modified Tyrode’s solution used in our previous publications (14, 17, 25). The solution contained (in mM), 135 NaCl, 5 KCl, 0.33 NaH_2_PO_4_, 5 Na-Pyruvate, 10 HEPES, 1 MgCl_2_, and 2 CaCl_2_ (pH 7.4) and is referred to as normal Tyrode’s solution (NT). For all experiments, NT with 5 mM glucose was considered as the control glucose concentration as it is within the physiological fasting range (NICE guideline PH38). This is a lower concentration of glucose than is traditionally used for these kind of experiments, often 11.1 mM, however, our previous data suggests alterations in functional parameters in cells at 10 mM glucose; a concentration would be considered as hyperglycaemic *in vivo* (14). Clinically, hyperglycaemia is defined as a blood glucose concentration above 11 mM (NICE guideline [CG130]). To investigate the effects of raised glucose, NT solutions containing 20 mM glucose were used, and mannitol (5 mM glucose with 15 mM mannitol) was used to ensure all normoglycaemic Tyrode’s solutions were osmotically balanced. This 20 mM glucose solution is referred to as “high glucose” for simplicity in the text. This glucose concentration, although well into the pathophysiological range, has been shown to be relevant to real clinical scenarios during MI (3, 17).

Nominally Ca^2+^ free solution used during the isolation of cardiomyocytes was as outlined above with no added Ca^2+^. Substrate-free 2 mM Ca^2+^ Tyrode’s solution (SFT) contained (in mM), 140 NaCl, 5 KCl, 0.33 NaH_2_PO_4_, 10 HEPES, 20 mM sucrose, 1 MgCl_2_, and 2 CaCl_2_ (pH 7.4). The pipette solution for whole cell electrophysiological recording contained (in mM), 30 KOH, 5 EGTA, 110 KCl, 10 HEPES, 1 MgCl_2_, 0.61 CaCl_2_, 1 ATP, 0.1 ADP, and 0.1 GTP (pH 7.2). Pipette solution for cell attached patch recording contained (in mM), 140 KCl, 10 HEPES, 1 CaCl_2_, and 0.5 MgCl_2_ (pH 7.4).

### Isolation of cardiomyocytes

Adult male Wistar rats (300–350 g) were killed by concussion and cervical dislocation. The protocol for the isolation of cardiomyocytes was as described previously (14, 17, 25, 29). Briefly, the heart was rapidly excised and placed into cold nominally Ca^2+^-free NT solution. The heart was then cannulated via the aorta on Langendorff apparatus and warmed (37°C) Ca^2+^-free NT was perfused in a retrograde manner via the aorta to clear residual blood. Following 6 min of perfusion, the solution was exchanged, and the heart perfused with Ca^2+^-free NT containing 0.66 mg/ml collagenase (Type II, Worthington’s, Lakewood, NJ, United States), 0.5 mg/ml protease (type XIV 15% Ca^2+^, Sigma-Aldrich, Gillingham, United Kingdom) and 1.93 mg/ml BSA prepared from factor V albumin (Sigma-Aldrich, Gillingham, United Kingdom) for 4–7 min until identification of rod-shaped cardiomyocytes in the perfusate. The solution was then exchanged for nominally Ca^2+^-free solution for 2 min, the heart cut down and the tissue washed three times with 2 mM Ca^2+^-containing NT. Cardiomyocytes were then mechanically dispersed from the tissue in a shaking water bath. Typically, this method yielded 70–90% rod-shaped cardiomyocytes and were stored in NT at room temperature in 60 mm culture dishes on a rocking platform to prevent pelleting of the cells. Cells isolated with this method are viable for up to 18 h following isolation with no detriment to contractility. Cells were typically used within 9 h for this study.

### Cell culture

The human ventricular cell line AC16 cells (Sigma-Aldrich, Gillingham, United Kingdom) were maintained and cultured in low glucose (5 mM) Dulbecco’s Modified Eagle’s Medium (DMEM) (Sigma-Aldrich, Gillingham, United Kingdom) supplemented with 10% foetal bovine serum (FBS) (Sigma-Aldrich, Gillingham, United Kingdom), 4 mM glutamine (Sigma-Aldrich, Gillingham, United Kingdom) and penicillin (100 IU)/streptomycin (100 mg/ml) (Sigma-Aldrich, Gillingham, United Kingdom) at 37°C under a humidified 5% CO_2_ atmosphere. Cells were used up to passage 20.

### Adenosine triphosphate-lactate dehydrogenase-protein assay

Cells were seeded at 10,000 cells/ml on standard 96-well tissue culture plate. The growth media was removed from the wells, cells were washed twice with Hank’s Balanced Salt Solution (HBSS) and replaced with high glucose (20 mM) DMEM with above supplements for 5, 15, or 30 min. Following glucose incubation, media was removed and saved for evaluation and AC16 cells were lysed in somatic cell ATP releasing reagent (Sigma-Aldrich, Gillingham, United Kingdom). Aliquots of the same lysate were then used to determine the ATP and protein level of the each well using the CellTiter-Glo Luminescent Cell Viability Assay (Promega, Southampton, United Kingdom) and BCA protein assay (Pierce, Thermofisher, Altrincham, United Kingdom), respectively, according to manufacturer’s guidelines. The lactate dehydrogenase (LDH) content of the lysate and media from the same well were measured according to the instructions of Cytotoxicity Detection Kit (Roche Diagnostics, Welwyn Garden City, United Kingdom) (30).

### Contractile function model

**FIGURE 1:**
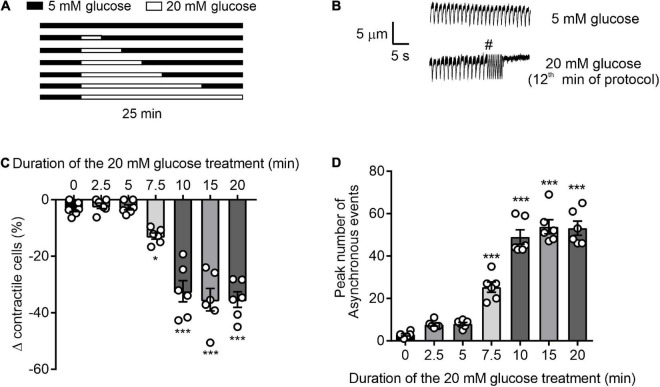
Time course of glucose-induced dysregulation of cardiomyocyte contractile function. **(A)** Illustration of the experimental design used in this figure. **(B)** Example traces showing cardiomyocyte contractions, initiated by 1 Hz EFS, in the presence of 5 or 20 mM glucose. Cardiomyocytes that contracted outside of the 1 Hz EFS, as demonstrated by **#** in panel **B**, or failed to contract were not defined as contractile cells. Therefore, only cardiomyocytes that contracted in time with 1 Hz EFS were counted as contractile cells. **(C)** Bar chart showing the mean percentage change in contractile cells (*p < 0.05, ***p < 0.001. One Way ANOVA with Holm-Sidak post-test). **(D)** Bar chart showing the mean number of arrhythmic episodes in each data set. ***p < 0.001 One-Way ANOVA with Holm-Sidak post-test. *n* = 6 experiments from six animals (>98 cells for each data set).

Cardiomyocytes were perfused at a rate of 5 ml/min at 32 ± 2°C and were stimulated to contract via electric field stimulation (EFS) at a rate of 1 Hz Digitimer DSXX (Welwyn Garden City, United Kingdom). The term “contractile cells” is used to indicate the percentage, or number, of rod-shaped cardiomyocytes contracting in rhythm with the EFS. Contractile function was observed via a JVC CCTV camera (JVC, Yokohama, Kanagawa, Japan) and recorded to DVD Panasonic DVD recorder (Panasonic, Kadoma, Osaka, Japan). Cells were perfused for 5 min with NT solution followed by 2.5–20 min perfusion with 20 mM glucose NT as indicated in Figure 1. The percentage of rod-shaped cardiomyocytes were counted every 30 s and used to form the time course (Figure 1A). The number of asynchronous contractions, contractions that occur in addition to the EFS and last for longer than 2 s in duration, were also counted. These asynchronous events could lead to the cell failing to contract as displayed in Figures 1A,B (14).

### Metabolic inhibition and reperfusion model

Using the same apparatus as above, cardiomyocytes were subjected to a metabolic inhibition and reperfusion (MI/R) protocol which consisted of 3 min perfusion with NT, 7 min of perfusion with a substrate-free metabolic inhibition Tyrode’s solution (SFT-MI) and 10 min washout with EFS at 1 Hz throughout. SFT-MI contained 2 mM Na-cyanide and 1 mM iodoacetic acid to block oxidative phosphorylation and glycolysis, respectively. The time to contractile failure, contractile recovery, and cell survival (as measured using Trypan blue exclusion) were noted for each cell. A contractile recovery of between 25 and 30% was indicative of a normal contractile recovery for control cardiomyocytes with anything above 45% contractile recovery representing cardioprotection (25). Modifications to this protocol, such as pre-treatment and changes to the washout protocol, are indicated in the figures and text.

### Patch clamp recording

Metabolic inhibition-activated currents were recorded in both whole cell and cell-attached patch configurations. Whole cell recordings were made from isolated cardiomyocytes using an Axopatch 200B amplifier, digitised using a Digidata 1440 and recorded and analysed using pCLAMP10.7 software (RRID:SCR_011323, Axon instruments, Scientifica, Uckfield, United Kingdom). Cardiomyocytes were perfused with NT solution at 5 ml/min at 32 ± 2°C with the membrane voltage held at 0 mV to inactivate all voltage-gated currents and to avoid the inward rectifier (IK_1_) current. Application of SFT-MI solution caused an activation of a metabolic inhibition-sensitive outward current attributed to the cardiac K_ATP_ current. The time to activation of this current was recorded and compared to that in cell pre-treated with a 20 mM glucose NT pre-treatment. Cardiac K_ATP_ (Kir6.2/SUR2A) current activation was also measured directly using cell-attached patch recording, with K_ATP_ being readily identified by its single channel current. Cell attached patches of membrane were held at 40 mV (an equivalent of ∼−110 mV from the sum of the pipette holding potential and the approximated resting membrane potential of −75 mV). At this potential, assuming a Kir6.2/SUR2A single channel conductance of around 70–80 pS, the single channel current should be ∼10 pA for each level of opening.

### Fura-2 measurement of [Ca^2+^]_i_

**FIGURE 2:**
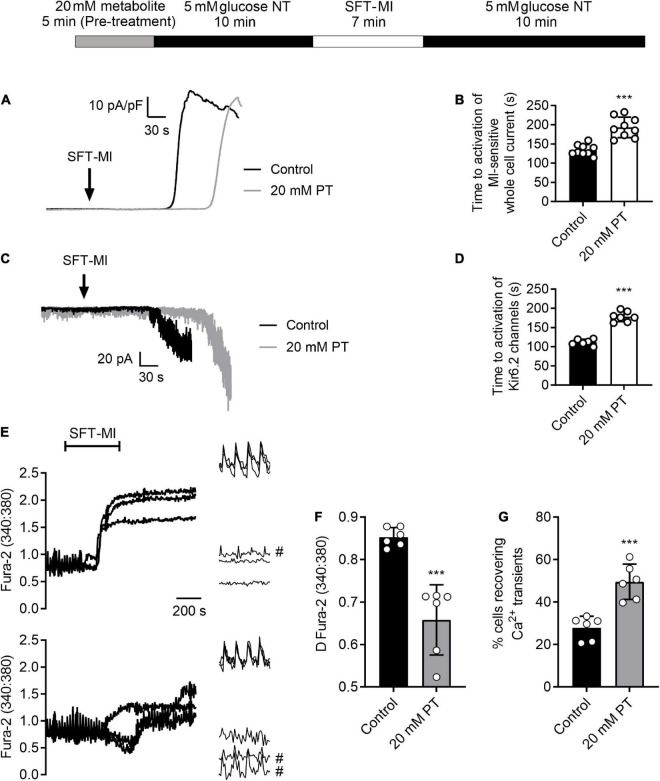
High glucose pre-treatment delays activation of the metabolic inhibition-sensitive K_ATP_ current and limits calcium overload during metabolic inhibition. **(A)** Example traces of whole-cell current recorded at 0 mV showing the activation of a metabolically-sensitive outward K^+^ current. **(B)** Bar chart showing the mean time to activation of this metabolic inhibition-sensitive current in control conditions (perfused for 15 min with NT prior to SFT-MI) or with 5 min high glucose pre-treatment. Mean time to activation was significantly delayed following 20 mM PT [***P > 0.001, Unpaired *T*-test, *n* = 9 (>14) for each group]. **(C)** Example traces of cell attached recording of sarcolemmal Kir6.2/SUR2A (IK_ATP_) current activation by metabolic inhibition, identified by its single channel conductance. **(D)** Bar chart showing the mean time to activation of Kir6.2/SUR2A channels with a burst duration longer than 250 ms. [***P > 0.001, Unpaired *T*-test, *n* = > 6 (>10) for each group]. **(E)** Example traces and expanded transients, recorded at the beginning and end of the protocol, from three cells in control conditions (perfused for 15 mins prior to SFT-MI with NT solution) and three cells in a 20 mM glucose pre-treated group (perfused for 5 min with 20 mM glucose NT followed by 10 min washout with NT). Cells recovering transients are marked with #. Cardiomyocytes were stimulated to contract using 1 Hz EFS and perfused at 34 ± 2°C. Fura-2 ratio images were taken once every 5 s to minimise photo bleaching. Transients were recorded at a rate of 12 ratios per second (24 images). **(F)** The mean change in Fura-2 ratio at the end of the 10 min simulated reperfusion period showing a reduction in Ca^2+^ accumulation in the 20 mM glucose pre-treated group compared to control conditions [***P < 0.001, unpaired *T*-test, *n* = 6 (>84 cells) per group]. **(G)** Mean percentage of cells recovering Ca^2+^ transients at the end of the simulated reperfusion period showing an increase following 20 mM glucose pre-treatment [***P < 0.001, unpaired *T*-test, *n* = 6 (>84 cells) per group].

Fura-2 measurements were made from cardiomyocytes as described previously (14, 17). Briefly, cardiomyocytes were loaded with 5 μM Fura-2 AM for 20 min at room temperature, then allowed to settle for a further 10 min in a heated perfusion chamber before being washed with NT solution prior to the start of the experiment. Cardiomyocytes were perfused at 5 ml/min at 32 ± 2°C and stimulated to contract at 1 Hz using electric field stimulation (EFS). Fluorescence was excited at alternately at 340 and 380 nm via a monochromator (PTI) and the emission collected above 520 nm using a Roper Cascade CCD camera (Photometrics, Tuscan, AZ, United States). Images were recorded using EasyRatioPro software (PTI, Birmingham, NJ, United States) and the data presented as the 340:380 ratio (Figure 2F), or a change in ratio (Figure 2G).

### Left anterior descending coronary artery ligation on a Langendorff system

The protocol for generation of an infarct using coronary ligation was previously described (17). Briefly, the heart was rapidly excised, placed in cold NT solution and then mounted on Langendorff apparatus via the aorta, perfused in a retrograde fashion and allowed to stabilise for 1 h and solutions bubbled with 100% O_2_ throughout. Any hearts that were not contractile (>60 bpm) following 1 h stabilisation or fell off of the canula at any part of the protocol, were excluded (2 of 29 hearts). The left anterior descending (LAD) coronary artery was ligated (40 min) to cause ischaemia using 5-0 USP braided silk suture and two pipette tips to form a reversible knot around the artery. The knot was then removed to start the 3-h reperfusion phase with the suture remaining in place to allow for re-ligation at the end of reperfusion. During all phases, temperature was maintained at 37°C by submerging the heart in Tyrode’s solution using a heated water jacket. Following re-ligation at the end of the protocol, Evans Blue dye (1% in Tyrode’s solution) was perfused through the heart, staining the areas unaffected by the ligation. The heart was then cut down from the Langendorff canula, wrapped in parafilm to prevent freeze-drying, and frozen for 1 h at −20°C. The heart was then cut into eight slices using a scalpel blade and stained in a Na_2_HPO_4_/NaH_2_PO4 solution (∼2:1 ratio of 0.1 M stock solutions until pH 7.4) with 10 mg/ml 2,3,5-triphenyltetrazolium chloride (Sigma Aldrich) to identify area at risk (AAR) and infarcted area (IA, white in appearance), respectively. To determine the AAR and IA, each slice was scanned on both sides and weighed. AAR and IA were calculated for each of the slices from the heart using ImageJ (RRID:SCR_003070). The AAR, IA and unaffected area sizes from ImageJ were then used to calculate the percentage infarct of the AAR by weight. Data were analysed by three researchers, including the experimenter. The two additional researchers were blinded to the conditions, and the mean of all three analyses were used for each heart.

### Protein kinase C inhibition

Tat-linked PKC inhibitor peptides were used at a concentration of 100 nM as described previously (14, 25, 31). Cardiomyocytes were preincubated with PKCα and β-specific Tat-linked inhibitor peptides for 15 min prior to the protocol outlined in each figure. PKCα and β were also pharmacologically inhibited in isolated cardiomyocytes using Gö6976 (300 nM: Tocris, Bristol, United Kingdom) and LY379196 (300 nM; a kind gift from Eli Lilly, IND, United States) which were perfused with the NT solutions as indicated in the figures. Equivalent concentrations of vehicle (DMSO) had no effect on cardiomyocyte function.

### Analysis software and statistics

All contractile function data was analysed in Microsoft Excel 2019 (RRID:SCR_016137). All patch clamp data was analysed in pCLAMP 10.7 (RRID:SCR_011323)^1^ software and Microsoft Excel 2019. Ca^2+^ fluorescence measurements were analysed using Winfluor V4.0.2 (J Dempster, University of Strathclyde)^2^ and Microsoft Excel 2019. Whole heart data was analysed using ImageJ V1.53^3^ and Microsoft Excel 2019. All graphs were made in GraphPad Prism V9.3 (RRID:SCR_002798)^4^ and statistical analysis performed in Prism 9.3 as indicated in the text. Data is reported as *n* = animals (cells) and bar charts presented with mean and standard deviation in solid bars with the individual mean per animal superimposed as circles. Data for AC16 cells is reported as *n* = experiments. Each experiment was the mean of >12 replicates for each condition. The statistical test(s), including post-tests, used for each data set are described in each figure legend along with the *n* number and the *P* value where appropriate.

## Results

### High (20 mM) glucose for greater than 7.5 minutes adversely impacts cardiomyocyte contractile function

To determine the duration of 20 mM glucose NT (high glucose) perfusion required to negatively impact contractile function, cardiomyocytes were exposed to high glucose for time intervals between 2.5 and 20 mins (Figure 1). In accordance with our previous findings (14), durations of high glucose perfusion ≥ 7.5 min reduced the proportion of rod-shaped cardiomyocytes maintaining contractile function to EFS and increased the number of asynchronous, ectopic contractions (Figures 1A–C). In cells with high glucose perfused for ≤5 min, there was no significant change in the percentage of contractile cardiomyocytes, or in the number of ectopic contractions. These data show that in isolated cardiomyocytes, exposure to high glucose for >7.5 min negatively impacts contractile function. Conversely, brief exposures to high levels of metabolic substrate, in the form of high glucose (5 min), did not alter contractile function.

### A short (5 minutes) high glucose challenge imparts cardioprotection against metabolic inhibition

A body of clinical work reports that elevated glucose at admission for ACS, irrespective of diabetic status, is deleterious to patient outcome (2, 3, 32–34). Furthermore, others have reported that active management of hyperglycaemia with insulin can improve patient outcomes (18–21). It was hypothesised that short (≤5 min) exposure to high glucose, to increase levels of glycolytic substrate, would impart cardioprotection against metabolic inhibition, akin to the short burst of ischaemia used to impart cardioprotection with ischaemic preconditioning (IPC).

**FIGURE 3:**
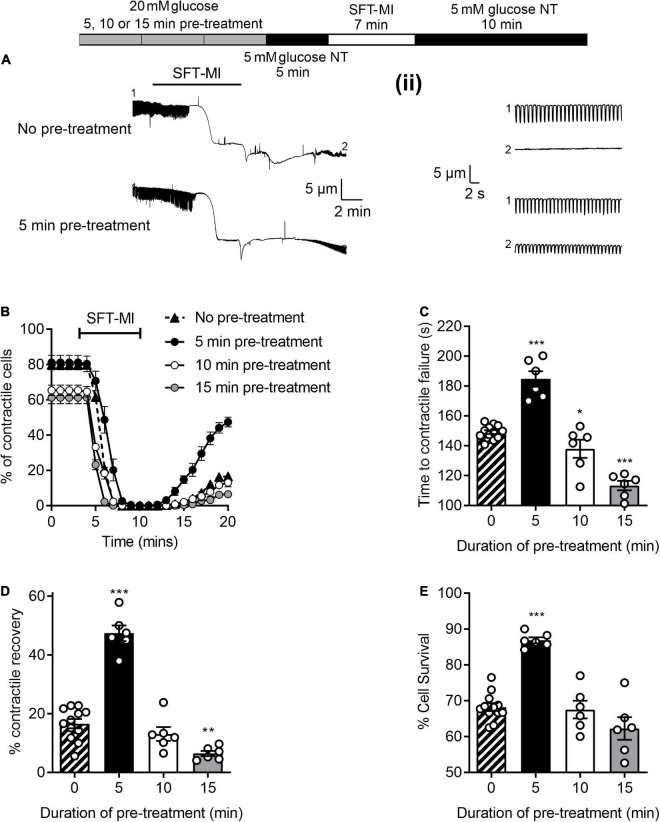
A short pre-treatment with elevated glucose imparts a cardioprotection, but longer durations reveal glucose-dependent toxicity. **(A)** Example video edge detection measurements from a cell in control (5 mM glucose) conditions and a cell following 5 min of pre-treatment with glucose prior to the MI/R protocol. **(ii)** Expanded traces showing 30 s of contractions from the beginning (1) and end (2) of the traces in panel **(A)**. **(B)** Time course showing the number of rod-shaped contractile cells from 3 min before the start of the metabolic inhibition in control (no pre-treatment), or following a 5, 10, or 15 min pre-treatment with 20 mM glucose NT. Bar charts showing **(C)** the mean time to contractile failure in metabolic inhibition, **(D)** the mean percentage of contractile recovery at the end of the MI/R protocol and **(E)** the mean percentage cell survival at the end of the MI/R protocol [**P < 0.01, ***P < 0.001, One-Way ANOVA with Holm-Sidak post-test, *n* = 6 (>131) cells for each group].

To investigate whether a short (5 min) high glucose challenge could impart cardioprotection against ischemia to isolated cardiomyocytes, the metabolic inhibition protocol was adapted to include a period of high glucose pre-treatment followed by a 5 min washout period with 5 mM glucose, as indicated in Figure 3. For this experiment, three durations of pre-treatment were used, 5, 10, and 15 min prior to the metabolic inhibition. With a 5 min high glucose pre-treatment there was a delay in the time to contractile failure (Figure 3C), increase in the percentage of cells recovering their contractile function (Figure 3D) and an increase in cell survival (Figure 3E); these are hallmarks of various established cardioprotective stimuli such as IPC and adenosine (25). With IPC, a delay in the time to contractile failure of ∼60 s is accompanied by increases of contractile recovery of ∼30% and cell survival of ∼15% are routinely recorded (25). A 10 min pre-treatment reduced the number of rod-shaped cardiomyocytes contractile at the start of the protocol (Figure 3A) but did not alter the other parameters measured (Figures 3B–D). Finally, a 15 min pre-treatment with high glucose was cardiotoxic as indicated by a reduced number of contractile cells (Figure 3A), shortened the time to contractile failure (Figure 3B) and fewer cardiomyocytes that recovered contractile function (Figure 3C). Together, these data suggest that a 5 min challenge with high glucose can impart cardioprotection against metabolic inhibition, however, longer exposures result in high glucose-dependent cardiotoxicity. These data suggest that prolonged exposure to enhanced glycolytic metabolite is cardiotoxic. Although this may be paradoxical to what might be expected, however, Supplementary Figure 1 shows that prolonged exposure to high glucose decreases ATP production in AC16 cardiomyocytes. Crucially, shorter duration of high glucose did not reduce ATP production. This could provide an explanation as to why prolonged exposure resulted in poor outcome in the simulated ischemia experiments.

### Cardioprotection imparted by a 5 minutes high glucose challenge is time interval and glycolytic metabolism dependent

**FIGURE 4:**
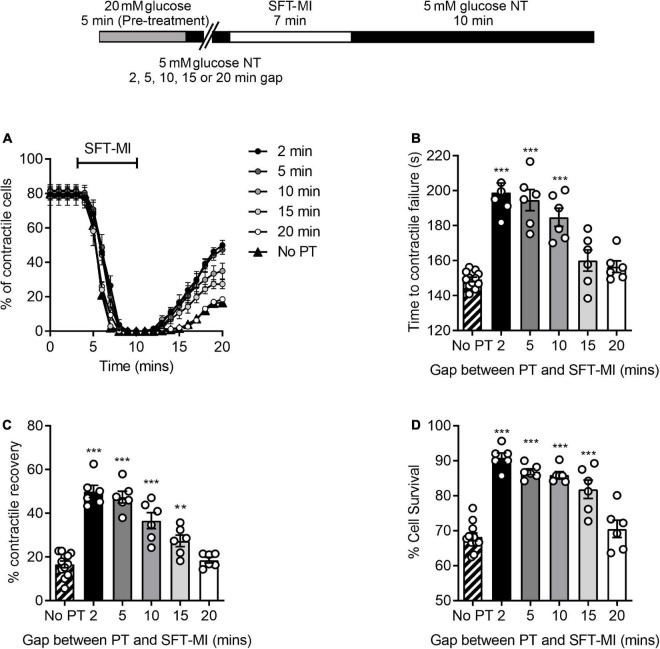
A 5 min pre-treatment with 20 mM glucose imparts a short-lived cardioprotection. **(A)** Time course showing the percentage of contractile cardiomyocytes throughout the metabolic inhibition protocol following a 5 min high glucose pre-treatment and a variable gap between the pre-treatment and metabolic insult. **(B)** Mean time to contractile failure, **(C)** mean percentage contractile recovery and **(D)** mean percentage of cell survival [**p < 0.01, ***p < 0.001, One-Way ANOVA with Holm-Sidak post-test. *n* = 6 experiments from six animals (>86 cells for each data set)].

It was hypothesised that the ischemic protection afforded to cells via a 5 min enhanced glycolytic load would decline by increasing the time interval between the high glucose challenge and metabolic inhibition. To investigate this, various time intervals (2–20 min) between high glucose pre-treatment and metabolic insult were included in the metabolic inhibition protocol, as indicated in the top of Figure 4. There was a marked delay in the time to contractile failure, an increase in contractile recovery and cell survival following a 5 min high glucose challenge with a 2-, 5-, or 10-min gap between the pre-treatment and the metabolic inhibition, however, this protection was largely lost by 15 min and completely lost with a 20 min gap (Figure 4). These data suggest a time interval-dependent, high glucose induced, cardioprotection. In further figures in this study, a 10 min gap between high glucose challenge and metabolic inhibition was used, as the protection afforded was significantly greater than control conditions but not at the potential upper limit of the protection (as seen with a 2- or 5-min gap).

**FIGURE 5:**
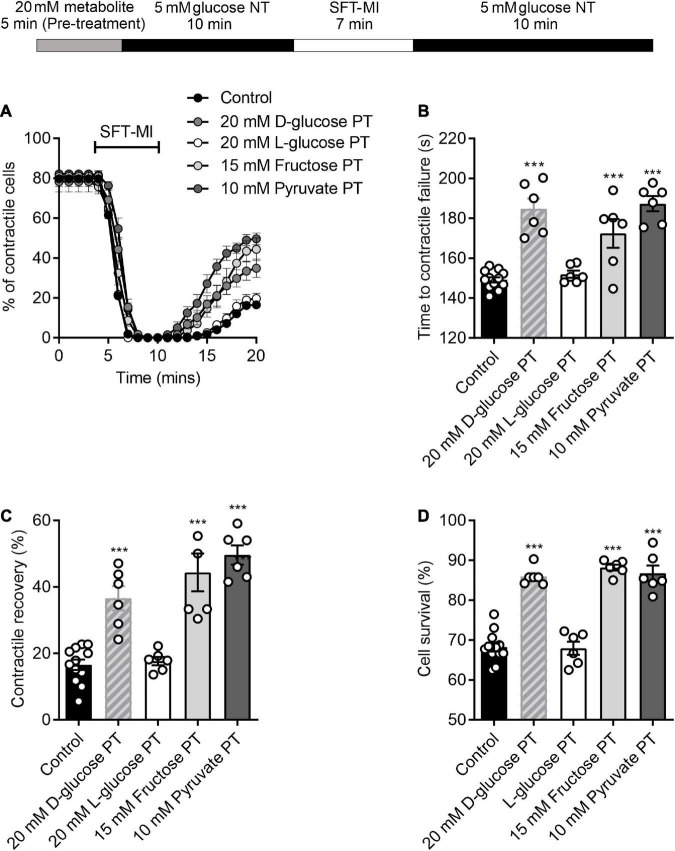
Protection afforded by high glucose pre-treatment can be mimicked with other glycolytic metabolites. **(A)** Example time course showing the percentage of contractile cells during the MI/R protocol following 5 min pre-treatment with 20 mM glucose, 15 mM fructose, 10 mM pyruvate, or 20 mM L-glucose. **(B)** Bar chart showing mean time to contractile failure. All pre-treatments show a significant delay in the time to contractile failure, except for L-glucose, which is a non-metabolised enantiomer [**P < 0.01, ***P < 0.001, One-Way ANOVA with Holm-Sidak post-test, *n* = 6 (>98 cells) for each group]. Bar charts showing **(C)** the percentage contractile recovery and **(D)** cell survival at the end of the MI/R protocol. In each case there is a significant increase in contractile recovery, except for the L-glucose pre-treated group [***P < 0.001, One-Way ANOVA with Holm-Sidak post-test, *n* = 6 (>102 cells) for each group].

To establish whether this protection was also triggered by alterations of glycolytic metabolism, the protocol was carried out with challenges with non-metabolised and metabolised sugars. A pre-treatment with 20 mM L-glucose, a non-metabolised enantiomer of D-glucose, showed no difference in the time to contractile failure, the contractile recovery or the cell survival compared to no pre-treatment control. This contrasted with the 20 mM D-glucose pre-treatment where there was a significant increase in all three measurements of cardioprotection (Figure 5). In addition, exchanging the 20 mM glucose pre-treatment for a 15 mM fructose with 5 mM glucose also imparted a cardioprotected-like phenotype, as did increasing pyruvate to 10 mM, with a significant increase in the time to contractile failure, increased contractile recovery and cell survival (Figure 5). These data suggest that enhanced glycolytic metabolism is responsible for the protective effect of a short period of high glucose pre-treatment.

### A 5-minute high glucose challenge delays K_ATP_ channel activation and improves Ca^2+^ handling

Our previous reports show that cardioprotective stimuli delays ATP depletion and thereby delays the time to downstream activation of the sarcolemmal cardiac K_ATP_ current (25). Comparable to cardioprotective stimuli, a 5 min high glucose pre-treatment delayed the time to activation of the metabolic inhibition-activated whole-cell current (Figures 2A,B) and Kir6.2/SUR2A sarcolemmal K_ATP_ current, measured using cell-attached recording (Figures 2C,D), in keeping with a hypothesized delayed ATP depletion.

Additionally, cardioprotective stimuli reduce the accumulation of intracellular Ca^2+^ during metabolic inhibition, and into reperfusion (25, 35–38). Fluorescence imaging was used to determine whether elevation of intracellular Ca^2+^ during metabolic inhibition and reperfusion can be attenuated with high glucose pre-treatment used as a cardioprotective stimuli. Figure 2E shows example traces in control conditions and following high glucose pre-treatment, showing a significant reduction in the end Fura-2 ratio and the change in Fura-2 ratio following pre-treatment (Figure 2F). Similar to the contractile recovery data in Figure 5 and Supplementary Figure 1, the recovery of Ca^2+^ transients at the end of the recording period were also increased in 20 mM glucose pre-treated cardiomyocytes (Figure 2G). Combined, these data show that short high glucose challenges display characteristics of established cardioprotective stimuli such as IPC.

### A key role for selective protein kinase C isoforms in the damaging effects of glucose

**FIGURE 6:**
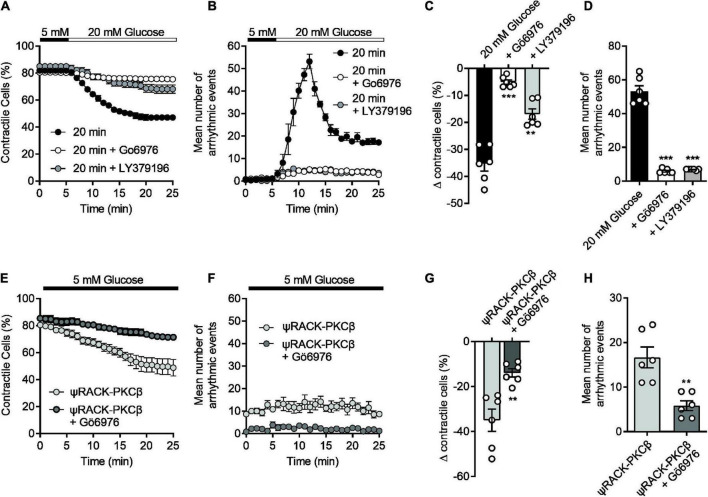
The high glucose-induced disruption of contractile function in isolated cardiomyocytes can be reversed with selective PKCα/β inhibition and mimicked with a PKCβ activator. **(A)** Time course showing the loss of contractile cardiomyocytes over a 20 min exposure to 20 mM glucose in the absence (black circles) and presence (white circles, Gö6976, grey circles, LY379196) of PKCα/β inhibitors. **(B)** Time course showing the number of contractions outside of the 1 Hz EFS in the cardiomyocytes recorded in panel **(A)**. **(C)** The mean change in contractile cells at the end of the protocol and **(D)** the mean peak number of arrhythmic evens in each condition (***P < 0.001, **P < 0.01, One Way ANOVA with Holm-Sidak post-test, *n* = 6 experiments from six animals, >123 cells for each group). **(E)** Time course showing the loss of contractile function in cardiomyocytes pre-incubated for 15 min with Tat-ψRACK-PKCβ-activating peptide in the absence (light grey) and presence (dark grey) Gö6976 in 5 mM glucose. **(F)** Time course showing the number of contractions outside of the 1 Hz EFS in the cardiomyocytes recorded in panel **(E)**. **(G)** The mean change in contractile cells at the end of the protocol and **(H)** the mean peak number of arrhythmic evens in each condition [**P < 0.0026 (G), **P < 0.0018 **(H)**, unpaired *t*-test, *n* = 6 experiments from six animals, >105 cells for each group].

Our previous publications (14, 16, 17), and others (13, 39) all suggest a link to protein kinase C (PKC) in the damaging effects of elevated glucose in cardiomyocytes. In particular, we have shown that glucose activates PKCα and β to increase cellular excitability leading to aberrant Ca^2+^ release events and changes in action potential morphology (14) which causes more damage following an ischaemic challenge (17). The experiments outlined in Figure 1 showed that increasing durations of high glucose perfusion increased the arrhythmic events seen in cardiomyocytes. This pro-arrhythmicity, seemingly triggered by a high glucose challenge, was largely reversed following pre-treatment with either 300 nM Gö6976 (PKCα and β inhibitor) or 100 nM LY379196 (PKCβ inhibitor), further confirming our previous findings of an involvement of PKCα and β (Figures 6A–D) (14, 17).

In cardiomyocytes pre-incubated with a cell permeant, tat-peptide linked, ψRACK-PKCβ-specific activating peptide, there was a similar increase in cardiac cell pro-arrhythmicity, in the absence of high glucose, that was reversed by treatment with Gö6976 (Figures 6E–H). Furthermore, use of the PKCβ activating peptide in metabolic inhibition experiments also reduced contractile recovery and cell survival analogous to treatment with high glucose, and was restored to control levels with selective PKCα/β inhibition (Supplementary Figure 2). These data suggest that we can mimic the damaging effects of a high glucose challenge with specific PKCβ activation.

### PKCα and β inhibitors further improve the 5-minute high glucose challenge-induced cardioprotection

**FIGURE 7:**
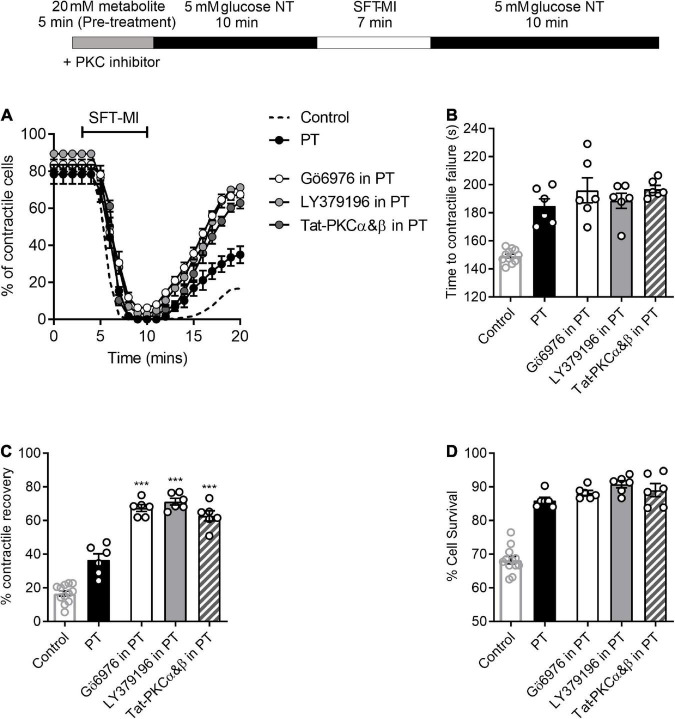
PKCα and/or β-selective inhibitors enhance the protected phenotype imparted by short high glucose pre-treatment. **(A)** Time course showing the percentage of contractile cardiomyocyte throughout the metabolic inhibition protocol following high glucose pre-treatment with or without PKC inhibitors, as indicated. **(B)** Bar chart showing the mean time to contractile failure, **(C)** mean percentage contractile recovery and **(D)** mean percentage of cell survival following metabolic inhibition in the absence and presence of selective PKC inhibition and with or without pre-treatment [***p < 0.001, One Way ANOVA with Holm-Sidak post-test. *n* = > 6 animals (>83 cells) for each data set. Each data set is compared to PT in the absence of PKC inhibitor. Control (no PT) data shown for comparison].

Although there were no obvious deleterious effects of a 5 min pre-treatment with 20 mM glucose (Figures 1A,C), it is clear from the data in Figures 1, 3, and our previous publications (11, 14, 16, 17), that deleterious effects occur when pre-treatments longer than 7.5 min are used. Given our previous findings, various selective PKCαβ inhibitors were used in combination with a 5 min high glucose challenge to determine whether a larger degree of cardioprotection against metabolic inhibition could be observed. Either small-molecule inhibitors, Go6976 (PKCα and β, 300 nM), LY379196 (PKCβ, 100 nM), or cell permeant Tat-peptide linked PKCα and β inhibitor peptides (50 nM of each peptide) were included in the high glucose pre-treatment. The PKC inhibitors had no effect on the outcome of the MI/R protocol in the absence of a high glucose stimulus (Supplementary Figure 3). In all cases where PKCα and/or β were inhibited along with the high glucose pre-treatment, contractile recovery was increased compared to high glucose pre-treatment alone, indicative of greater cardioprotection (Figure 7). There was, however, no further delay in the time to contractile failure or further increase in the percentage of cells surviving the MI/R protocol (Figure 7). In keeping with our previous findings, selective PKCα/β inhibition did not affect the tested parameters in 5 mM glucose with no high glucose challenge (Figure 7).

Protein kinase Cε is conventionally thought to be part of the cardioprotective pathway, where selective inhibition of this isoform removes protection afforded by many protective stimuli (25, 40, 41). 20 mM D-glucose-induced protection was unaffected by selective PKCε inhibition with a cell permeant Tat peptide-linked PKCε inhibitor peptide with either a 5- or 10-min gap between glucose and metabolic inhibition (Supplementary Figure 4). This peptide has previously been shown, in our hands, to abolish cardioprotection afforded by a number of stimuli (14, 25). These findings suggest that the short pre-treatment with 20 mM glucose was not causing protection by activation of PKCε, therefore making this distinct from other conventional cardioprotective interventions.

### PKCα/β inhibitor Gö6976, together with high glucose challenge, reduces infarct size in the *ex vivo* whole heart coronary ligation model

**FIGURE 8:**
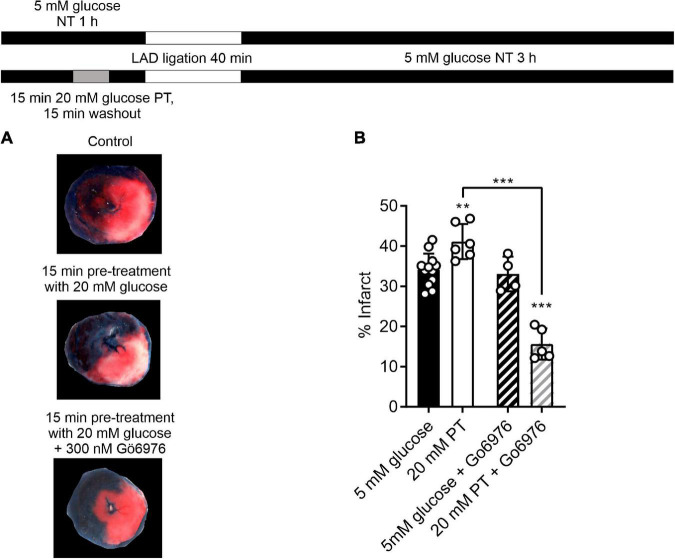
Selective inhibition of PKCα/β with Gö6976 reveals a 20 mM glucose pre-treatment dependent reduction in infarct size. **(A)** Example images from a control heart, heart following an amended protocol using a 15-min pre-treatment with 20 mM glucose and a heart following 15-min pre-treatment with 20 mM glucose in the presence of 300 nM Gö6976. The experimental protocol is shown above. **(B)** Bar chart showing the mean percentage infarct by weight of the different groups. Circles show data from individual hearts (**P < 0.01 and ***P < 0.001 Two-way ANOVA with Tukey’s post-test, *n* = 12, 6, 4, and 5 hearts for control, 20 mM PT, 5 mM glucose with Gö6976 and 20 mM PT with Gö6976 groups). There was no difference in the effectiveness of coronary blockade in the 4 groups measured as the percentage area at risk (41.9 ± 4.5, 43.2 ± 4.5, 39.4 ± 2.3, and 38.9 ± 2.6% (mean ± S.D., *n* = 12, 6, 4, and 5 hearts for control, 20 mM PT, 5 mM glucose with Gö6976 and 20 mM PT with Gö6976 groups, Two-way ANOVA).

Having demonstrated that selective PKCα/β inhibition enhanced the protective effects of a short pre-treatment with high glucose in isolated cells, a similar protocol was used in the whole heart in a left anterior descending (LAD) coronary artery ligation model. A 5-min pre-treatment with glucose, with or without Gö6976, had no significant effect on the mean infarct size [34.1 ± 1.3, 31.5 ± 1.9, and 33.4 ± 3% in control (*n* = 11), 5 min pre-treatment (*n* = 4) and 5 min pre-treatment with 300 nM Gö6976 (*n* = 4)]. This may be because different durations of substrate perfusion are required for whole organ systems such as the heart compared to experiments on isolated cardiomyocytes, which allow almost instant changes in solution to be performed. The duration of the high glucose pre-treatment was increased to 15 min, which caused a detectable increase the infarct size [from 34.1 ± 1.3% in control conditions to 41 ± 2.3% (*n* = 6) following 15 min pre-treatment with high glucose (Figures 8A,B)]. Co-treatment with Gö6976, however, markedly reduced the infarct following high glucose challenge from 34.1 ± 1.3 to 15.6 ± 1.7%. This reduction in infarct size was not seen in hearts treated with Gö6976 in the absence of a high glucose pre-treatment (33.1 ± 2.3%, *n* = 4; Figure 8B). These data show that in whole-heart *ex vivo* experiments, a high glucose challenge for 15-min increased infarct size that was reversed with PKCα/β inhibition, further demonstrating a PKC-dependence to the modulation.

## Discussion

This study demonstrates that a short 5-min challenge with increased extracellular metabolic substrate imparted a cardioprotective phenotype to cardiomyocytes that was short-lived in these experimental models but gave a protection that was as substantive as well-established methods of cardioprotection, such as ischaemic preconditioning (14, 25). The protection afforded by the elevated metabolic substrate was further enhanced by co-treatment with selective PKCα and/or β inhibition, similar to our previous findings (11, 14, 17, 25). These findings suggest that we should be giving consideration of the damage that glucose could be doing not just in terms of weeks, but rather over the duration of minutes.

Evidence supports this with Kir6.2 openings and whole cell current. Although short burst of glucose did not increase ATP levels in cells (Supplementary Figure 1), the time for Kir6.2 opening was delayed thereby demonstrating ATP was preserved for longer; a hallmark of established cardioprotective stimuli, such as IPC (25). We suggest that the 5 min pre-treatment used in the cellular section of this study was not long enough to fully activate the deleterious, PKCα/β-dependent cardiotoxicity.

This current report results demonstrates a critical time-dependent effect, whereby glucose is cardiotoxic to cardiomyocytes with greater than 7.5 min exposure but protective with less than 5 min, suggesting a temporal switch between protection and toxicity. Although more clinical research is needed to determine whether this role translates to the clinical scenario, it does fit with the clinical data reporting elevated admission glucose in ACS patients is associated with poor outcome. Such time-dependence does, however, offer a plausible explanation to the conflicting reports on the benefit of insulin infusion to reduce glucose in ACS patients (42–44), together with the conflicting reports of benefit with glucose-inulin-potassium infusion (18–21). It is plausible that the benefit of this therapy may be time-dependent and that the control of glycaemia in patients in more effective with an early intervention. A greater appreciation of this time-dependent effect is also required for basic research, where acute increases of extracellular glucose has been reported to be cardiotoxic to cardiomyocytes by our lab (14, 17), and others (13, 15, 45–51). Furthermore, we have also demonstrated this in whole heart *ex vivo* measurements of infarct size both in this and our previous manuscript (17).

The concentration of glucose used in this study, 5 mM as our “normoglycaemic”, is no unique amongst researchers, however, 11.1 mM is often used and 25 mM may be used in culture media. Although these elevated concentrations are used elsewhere to compensate for other metabolic substrates that might be found *in vivo*, our studies have previously shown that even 10 mM glucose gives rise to differences in cardiac cell function and response to conditioning stimuli (14). We see no detriment in cellular or whole heart recordings using 5 mM glucose as our normoglycaemic control, and advocate this concentration as an appropriate physiologically normal glucose (14, 17, 25).

The complex relationship between glucose, time and myocardial damage has also been reported with other metabolites. Non-obesogenic mice fed a high-fat diet exhibit increased susceptibility to myocardial reperfusion injury (49), however, switching back to a normal diet before cardiac insult reduced cardiac vulnerability (48). Ethanol has also been reported to have a direct cardioprotective effect on the I/R challenged myocardium (26), and similar to our glucose work described here, ethanol can prevent its own protection if it is not washed out or sufficiently metabolized prior to the onset of ischemia (27). Combined, these reports suggest that acute exposure to elevated levels of metabolite, somewhat paradoxically, result in greater damage during metabolic inhibition. Although the reasons behind this require further research, we suggest that, for glucose at least, the damage be in part due to the build-up of metabolites that are forced into diacylglycerol synthesis, which then cause aberrant activation of DAG-sensitive PKC isoforms (13, 52, 53).

A noticeable difference that we can report between the ethanol-dependent and glucose-dependent cardioprotection is the PKC isoforms that are involved. Both ethanol and arachidonic acid (AA) have been shown to activate PKCε (26, 46, 54, 55), activation of which is well established to impart cardioprotection (56, 57). However, the use of a Tat-PKCε inhibitor peptide did not alter the cardioprotective effect of glucose pre-treatment (Supplementary Figure 4). The use of selective PKCα/β inhibitors further enhanced the cardioprotection imparted by glucose pre-treatment, indicating PKCα/β activation within minutes can cause deleterious effect to cell function (Figure 7).

Previous experiments using acute hyperglycaemia in animal models has shown an increased infarct size and reduced functional recovery (14, 44, 58, 59), therefore it is difficult to imagine a scenario where clinicians would briefly *introduce* hyperglycaemia as a cardioprotective therapy. There is a growing body of clinical evidence that demonstrates that glucose, irrespective of diabetic status, is associated with poor outcome for patients with myocardial infarction (3, 8, 34, 51, 60, 61). This damaging outcome combined with the large proportion of patients that have elevated glucose, perhaps though stress-induced hyperglycaemia (3, 17, 62), outlines the need to prevent the harmful effects of glucose in ACS. What is compelling, and evidenced in our previous publications (14, 16, 17), is that acute elevations of glucose coupled with PKCαβ inhibition mask the damaging effects of glucose, and so reveals a glucose metabolism-dependent protective effect. This was evident in both isolated cell models, and in whole heart coronary ligation experiments. These findings raise the possibility that PKCα/β inhibition may provide a novel therapy to confer cardioprotection in ACS patients with hyperglycaemia, whilst preventing the risk of causing hypoglycaemia by glucose lowering interventions. Indeed, PKC inhibitors of the isoforms identified to play a role in glucose-induced cardiac dysregulation are already in use in clinical trials. The PKCβ inhibitor, Ruboxistaurin, an LY379196 analogue, is currently in phase I/II evaluation for treatment of heart failure (NCT02769611), however, is listed in this documentation as a PKCα inhibitor. In addition, various, pre-clinical studies have suggested PKCβ inhibition as a potential therapeutic target in heart failure via several mechanisms (63). Inhibition of PKCβ at the point of ischaemic damage may reduce the glucose-dependent exacerbation of this injury and also help attenuate the development of heart failure.

Findings that acute hyperglycaemia activates PKC to cause negative effects on cardiomyocyte function, may go in some part to explain this loss of efficacy in clinical trials compared to *in vitro* and *in vivo* animal model studies. Several groups have shown that cardioprotective stimuli fail to work in elevated glucose (50, 64–66). Our previous manuscripts in this area demonstrate that although cardioprotection can be abolished by elevated glucose, selective PKCα and β inhibition restored cardioprotection (14). PKCα/β inhibition therefore may provide a route for re-investigation of a number of failed therapeutics for imparting cardioprotection.

### Study limitations

Our data in this study was investigated in rat cardiomyocytes and whole heart using an *ex vivo* coronary ligation model. Further research in this area is required to determine the therapeutic potential of glucose challenge combined with selective PKC inhibition. However, the number of studies reporting deleterious effects of glucose on cellular functions and the mapping of this to the *in vivo* and clinical scenario is providing potentially interesting new avenues to explore. In our manuscripts, we have taken care to describe the effects of an *acute* treatment with high glucose and have not discussed what could happen with chronic hyperglycaemia as might be seen in a diabetic patient. The potential involvement of advanced glycation end products (AGE), ROS sympathetic drive and the polyol pathway have not been investigated in this manuscript given that they are often associated with longer term changes in chronic hyperglycaemia (52, 53, 67). Further to this, it should be noted that the experiments in this study do not include any free fatty acids in the solutions, the preferred metabolic substrate in cardiac cells (68). In our previous studies, we demonstrate that PKC was being activated not just functionally, but also showing changes in cellular distribution in response to high glucose (14, 17), but the mechanism behind this is unclear. We hypothesise that it involves *de novo* synthesis of diacylglycerol from a backup of glycolytic enzymes (52, 53, 69). All of our data to date has been carried out in otherwise healthy animal tissue with no known comorbidities. This is unlike the clinical scenario where a patient presenting with an AMI would be unlikely to have no other potential confounding factors. What is clear from our and others data is that glucose demonstrates a time-dependent cardiotoxicity that may well underlie damage to cardiomyocyte integrity and function in the setting of ischaemia. This suggests that the clinical association between hyperglycaemia and adverse outcome after ACS may indeed be the result of direct glucose-toxicity rather than an epiphenomenon.

## Conclusion

In summary, we have demonstrated in this manuscript that a short treatment/challenge with high glucose, or alternative metabolite, can impart a cardioprotected-like phenotype to both isolated cells and the whole heart. Such protection by elevated glucose requires the administration to be short restored to normal physiological levels. This protection is markedly improved by the co-administration of selective PKCα and β-selective inhibitors, which then has no time dependence to it, as the deleterious effects shown in our previous manuscripts are suppressed (14, 16, 17). Given that ∼48% of ACS patients showed a blood glucose greater than 8 mM (3, 17), indicating some degree of stress-hyperglycaemia, we suggest that selective PKCα/β inhibitors should be investigated as a potential cardioprotective alternative to aggressive lowering of blood glucose. Our findings suggest that this could turn a deleterious increase in glycaemia to a novel cardioprotective intervention.

## Data availability statement

The original contributions presented in this study are included in the article/Supplementary material, further inquiries can be directed to the corresponding authors.

## Ethics statement

The animal study was reviewed and approved by University of animal welfare ethical review board (AWERB_2018_44).

## Author contributions

SB: contributed to experimental design, acquired, analysed, interpreted the data, and drafted the manuscript. SE, MA, CM, and SM: acquired, analysed, interpreted the data, and helped with manuscript revision. MS: acquired, analysed, and interpreted the data. IS, PS, and AC: conception and design of the work, critically appraised, and revised the manuscript. RR: conception and design of the work, acquired, analysed, interpreted the data, critically appraised, and revised the manuscript. All authors approved the final version of manuscript, agreed to be accountable for all aspects of the work in ensuring that questions related to the accuracy or integrity of any part of the work are appropriately investigated and resolved. All work was carried out at the Universities of Liverpool or Leicester in RR’s laboratory.

## Footnotes

1
https://support.moleculardevices.com/s/article/Axon-pCLAMP-10-Electrophysiology-Data-Acquisition-Analysis-Software-Download-Page

2
http://spider.science.strath.ac.uk/sipbs/software_imaging.htm

3
https://imagej.nih.gov/ij/download.html

4
https://www.graphpad.com/scientific-software/prism/
