# Supplementary material for: Selective protein kinase C inhibition switches time-dependent glucose cardiotoxicity to cardioprotection
Source: Front Cardiovasc Med. 2022 Sep 7;9:997013. doi: 10.3389/fcvm.2022.997013 (PMC9489859; doi:10.3389/fcvm.2022.997013)
Supplement: Supplementary file 1 [file Data_Sheet_1.docx]

Supplementary Material


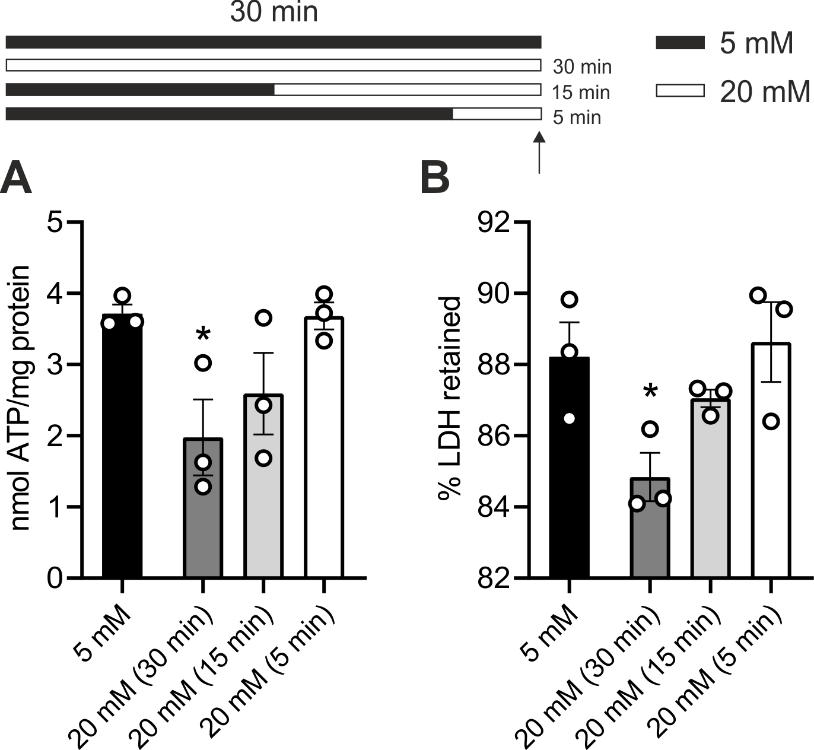


**Supplementary Figure 1**

**Increasing duration of treatment with high glucose in AC16 cells reduces cellular ATP production and increases cell toxicity**

**A,** mean data from ATP and LDH assays in control cells (5 mM glucose) or following 5-, 15- or 30-min treatment with high (20 mM) glucose. *p<0.05 One-Way ANOVA with Dunnett’s post-test. **B,** Mean data from LDH assay in control cells (5 mM glucose) or following 5-, 15- or 30-min treatment with high (20 mM) glucose. *p<0.05 One-Way ANOVA with Dunnett’s post-test.


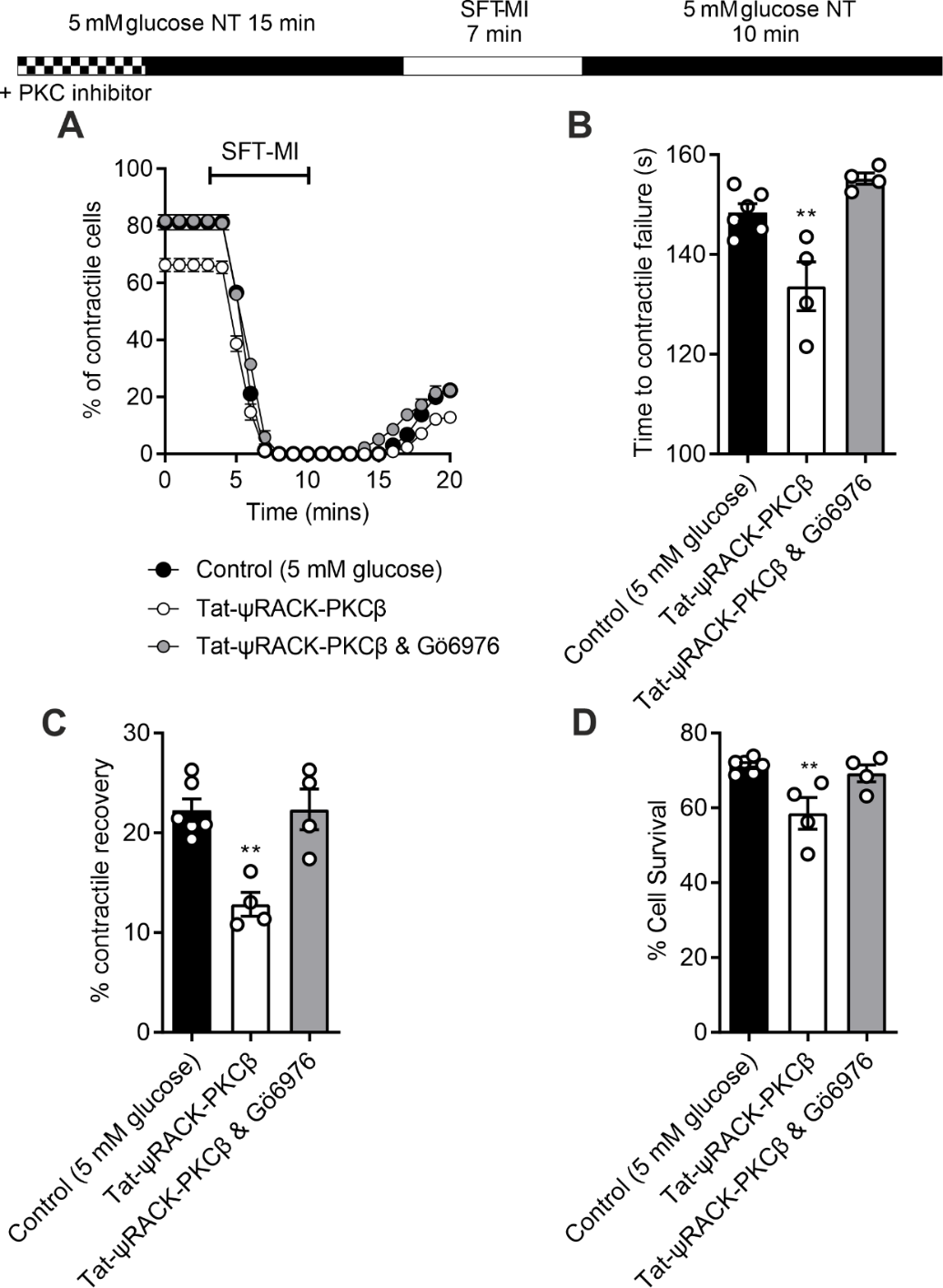


**Supplementary Figure 2:**

**Selective activation of PKCβ mimics the effects of high glucose on contractile function and recovery from metabolic inhibition.**

A, Time course showing the mean percentage of contractile cardiomyocytes during a metabolic inhibition and reperfusion protocol in control conditions (5 mM glucose, black circles), following 15 min pre-treatment with Tat-ψRACK-PKCβ-activating peptide in the absence (white circles) and presence (grey circles) of Gö6976 in 5 mM glucose (protocol shown above). Bar charts showing the mean time to contractile failure (B), percentage contractile recovery at the end of the protocol (C) and the cell survival as measured using Trypan blue (D). (**P<0.01 for each group, One Way ANOVA with Holm-Sidak post test, n = >4 experiments from 4 animals (>78 cells) for each experimental group).


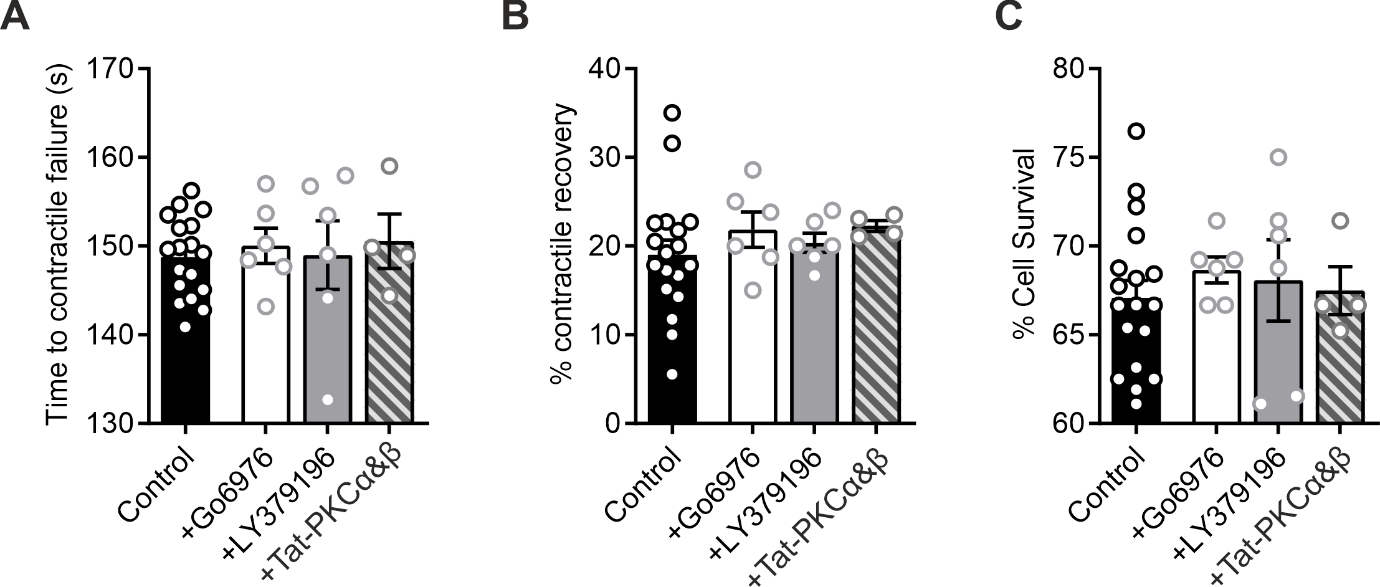


**Supplementary Figure 3**

**Selective PKCα and/or β inhibition had no effect on the outcome of the MI/R protocol in the absence of a high glucose challenge.**

Control cardiomyocytes, or cardiomyocytes pre-treated with Gö6976, LY379196 or with Tat-PKCα&β inhibitor peptides were exposed to the MI/R protocol in the absence of high glucose pre-treatment. There was no significant difference in the time to contractile failure **(A),** percentage of cell survival **(B)** or the percentage cell survival **(C)** following PKC inhibitor treatment.


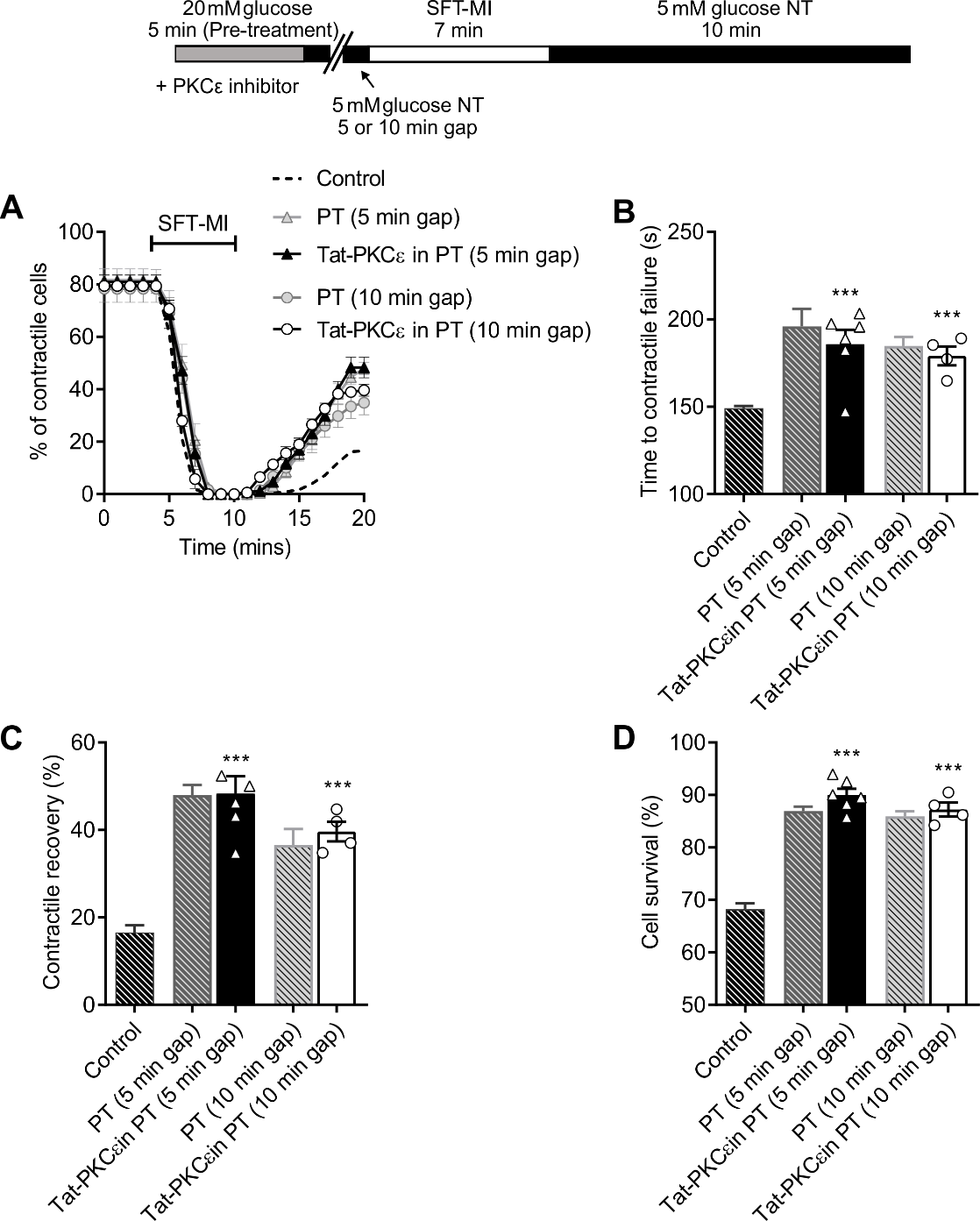


**Supplementary Figure 4**

**Selective inhibition of PKCε did not affect the protection afforded by high glucose pre-treatment**

A, time course showing the percentage of contractile cardiomyocyte throughout the simulated ischaemia protocol following high glucose pre-treatment for 5 min with a 5- or 10-min gap between challenge and metabolic inhibition, in the presence or absence of PKCε inhibition. B, bar chart showing the mean time to contractile failure, C, mean percentage contractile recovery and D, mean percentage of cell survival following simulated ischaemia. Control and pre-treatment data with a 5- or 10-min gap is reproduced from figure 3 and shown as hatched bars. Pre-treatments were significantly different to control, but there was no significant difference between the pre-treatments in the presence or absence of PKCε inhibition. (***p<0.001, Two-Way ANOVA with Holm-Sidak post-test. n = >4 (>94 cells for each data set)).
